# Efficacy of a *Limosilactobacillus reuteri* DSM 17938 and BG-R46^®^ Formulation in Predominantly Breastfed Infants with Infant Distress Syndrome: A Randomized Double-Blind Placebo-Controlled Trial

**DOI:** 10.3390/nu18213468

**Published:** 2026-10-22

**Authors:** Flavia Indrio, Luca Pecoraro, Pierluigi Manni, Valeria Verdesca, Antonio Di Mauro

**Affiliations:** 1 Department of Experimental Medicine, Pediatric Section, Medical School MedTech, University of Salento, 73100 Lecce, Italy; 2 Pediatric Unit, Ospedale Vito Fazzi, ASL Lecce, 73100 Lecce, Italy; 3 Pediatric Primary Care, National Pediatric Health Care System, ASL Lecce, 73100 Lecce, Italy; 4 Pediatric Primary Care, National Pediatric Health Care System, ASL Bari, 70100 Bari, Italy

**Keywords:** infantile colic, *Limosilactobacillus reuteri* DSM 17938, *Limosilactobacillus reuteri* BG-R46™, infant distress syndrome

## Abstract

*Background*: Infant distress syndrome, previously termed Infantile colic, is a common disorder of gut–brain interaction characterized by excessive crying and impaired behavioral regulation. Although *Limosilactobacillus reuteri* DSM 17938 has shown efficacy in predominantly breastfed infants, treatment responses remain variable, highlighting the need to investigate alternative probiotic formulations. BG-R46™, a strain derived from DSM 17938, exhibits distinct properties in preclinical models, providing a rationale for evaluating the combined formulation. This study investigated the efficacy and tolerability of DSM 17938 plus BG-R46™ compared with placebo in infants with infantile colic. *Methods*: In this randomized, double-blind, placebo-controlled trial, 50 infants were randomized to receive either the probiotic formulation or placebo for 21 days. Forty-eight infants (24 per group) were included in the intention-to-treat and per-protocol analyses. The primary endpoint was the change from baseline to Day 7 in total infant distress time, defined as the sum of awake fussing, crying, and inconsolable crying recorded using the Baby’s Day Diary^®^. Secondary outcomes included crying time, infant behavioral regulation (sleep duration, awake and content behavior, awake fussing, and inconsolable crying), maternal emotional well-being assessed using the Edinburgh Postnatal Depression Scale (EPDS), and safety. *Results*: Baseline infant distress time was comparable between groups. At Day 7, infant distress time decreased by 43% in the probiotic group versus 13% in the placebo group (−236.0 ± 111.5 vs. −66.7 ± 100.1 min/day; *p* < 0.001). At Day 21, reductions reached 62% and 26%, respectively. The probiotic formulation was well tolerated throughout the study. *Conclusions*: Supplementation with *L. reuteri* DSM 17938 plus *L. reuteri* BG-R46™ significantly reduced infant distress compared with placebo.

## 1. Introduction

Infantile colic, now renamed Infant distress syndrome is among the most common functional gastrointestinal disorders (FGIDs) during early infancy, affecting approximately 15–25% of otherwise healthy infants within the first months of life [1]. Although it is generally considered a self-limiting condition, excessive crying and fussing represent a substantial burden for families, contributing to parental anxiety, impaired quality of life, increased healthcare utilization, early discontinuation of breastfeeding, and, in severe cases, maternal depression and infant abuse [2]. Consequently, despite its benign natural history, infantile colic remains one of the leading reasons for pediatric consultations during early infancy.

The understanding of infantile colic has progressively evolved over the last decades. Historically, the Wessel “rule of threes” defined colic as crying lasting more than three hours per day, on more than three days per week, for at least three weeks.

Subsequently, the Rome III and Rome IV criteria recognized infantile colic as a functional gastrointestinal disorder characterized by recurrent and prolonged periods of crying, fussing, or irritability occurring without an identifiable cause in infants younger than five months [3].

More recently, the Rome V diagnostic criteria have further refined the concept of infantile colic as infantile distress syndrome and emphasized that excessive crying and fussing represent a disorder of gut–brain interaction rather than a disease defined solely by quantitative crying thresholds. Rome V highlights the importance of caregiver perception of inconsolable crying episodes, the absence of organic disease, and the significant impact on family functioning while acknowledging the limited clinical value of rigid time-based criteria in routine practice [4]. These updated criteria better reflect current understanding of infantile colic as a multifactorial condition involving complex interactions among gastrointestinal function, microbiota development, immune activation, visceral hypersensitivity, neurodevelopmental maturation, circadian regulation, and psychosocial factors [5].

Although its exact pathophysiology remains incompletely understood, increasing evidence supports a central role for alterations of the intestinal microbiome and low-grade intestinal inflammation [6]. Infants with colic have been shown to exhibit microbial dysbiosis characterized by reduced bacterial diversity, lower abundance of beneficial commensals, increased Proteobacteria, and elevated fecal calprotectin concentrations, suggesting the presence of intestinal immune activation [7,8,9,10]. These findings have generated growing interest in microbiota-targeted interventions aimed at restoring intestinal homeostasis during this critical developmental window [11].

Among probiotic interventions, *Limosilactobacillus reuteri* DSM 17938 is currently the best-studied strain for the management of infantile colic [12]. Multiple randomized controlled trials and subsequent meta-analyses have demonstrated that supplementation with *L. reuteri* DSM 17938 significantly reduces daily crying and fussing time, particularly in predominantly breastfed infants, leading several international scientific societies to recommend its use in this population [13].

Proposed mechanisms include modulation of gut microbial composition, reduction in intestinal inflammation, improvement of intestinal barrier function, regulation of gastrointestinal motility, attenuation of visceral pain signaling, and modulation of the microbiota–gut–brain axis [14,15,16].

Despite these encouraging results, clinical response to *L. reuteri* DSM 17938 is heterogeneous and appears to depend partly on feeding type and population [17].

Although improvement may be detectable within the first week in some trials, treatment effects are more consistently demonstrated after approximately 14–21 days [18]. Faster, predictable symptom relief remains an important unmet clinical need.

BG-R46™ is a proprietary *L. reuteri* strain derived from DSM 17938 through repeated stress adaptation while maintaining a closely related genomic background [19]. Preclinical studies suggest that BG-R46™ may enhance several biological functions associated with colic improvement, including increased adenosine metabolism [20], greater stimulation of endogenous melatonin production [21], preservation of epithelial barrier integrity, modulation of gastrointestinal motility, and regulation of nociceptive pathways [22]. Preclinical observations have suggested strain-specific differences in immunomodulatory activity, providing a mechanistic rationale for investigating the combination. However, the clinical relevance of these findings and any potential interaction between the two strains remain unproven. Furthermore, the activity of BG-R46™ was enhanced by human milk oligosaccharides, which are abundant in breast milk, further supporting its relevance in early-life nutrition [23]. Since adenosine represents a precursor of melatonin and melatonin has been implicated in gastrointestinal motility regulation and circadian maturation, these mechanisms may potentially contribute to a faster therapeutic response. However, clinical evidence evaluating BG-R46™ in infants with colic is currently lacking.

Building upon the existing evidence supporting *L. reuteri* DSM 17938 in breastfed infants with colic, the present study was designed to evaluate the clinical efficacy and tolerability of a formulation combining DSM 17938 with BG-R46™ compared with placebo. Although preclinical findings provide a biological rationale for investigating this combination, whether BG-R46™ offers additional clinical benefits beyond those of DSM 17938 alone remains to be established.

## 2. Materials and Methods

### 2.1. Trial Design

This was a prospective, double-blind, randomized, placebo-controlled, parallel-group clinical trial designed to evaluate the efficacy and safety of a probiotic combination containing *Limosilactobacillus reuteri* DSM 17938 and *L. reuteri* BG-R46™ in infants with infantile colic. Participants underwent a 7-day run-in period, followed by a 21-day intervention and a 7-day post-intervention follow-up. The study was conducted in accordance with the Declaration of Helsinki and Good Clinical Practice guidelines. The protocol was approved by the local Ethics Committee (Prot. 2138/CEL Comitato Etico Locale IRCCS Istituto Oncologico Puglia Salute), and written informed consent was obtained from either parents or legal guardians before enrolment.

The trial was prospectively registered at ClinicalTrials.gov (Identifier: NCT06462651) before enrolment of the first participant.

The study was conducted at the Pediatric Unit of Vito Fazzi Hospital of the University of Salento, Lecce, Italy. Potentially eligible infants were recruited from both outpatient referrals to the hospital pediatric clinic and routine consultations in primary care pediatric practices.

Eligibility was confirmed by a pediatrician before enrolment.

Recruitment was carried out between June 2025 and February 2026.

### 2.2. Participants

Healthy term infants aged between 3 and 12 weeks were eligible if they fulfilled the inclusion criteria, including the diagnostic criteria for infantile colic according to the Rome IV criteria applicable at the time of study initiation.

Diagnosis was confirmed following physician evaluation and prospective documentation of crying and fussing behavior using the Baby’s Day Diary^®^, with at least one 24-h recording demonstrating ≥3 h of crying and/or fussing during the run-in period.

Eligible infants were required to have been born between 37 + 0 and 42 + 0 weeks of gestation, to have an appropriate birth weight for gestational age, and to be considered healthy on physical examination.

Major exclusion criteria included severe gastroesophageal reflux disease, failure to thrive, fever, diarrhea, hematochezia, congenital systemic diseases, previous exposure to probiotics or systemic antibiotics within seven days before enrolment, or the use of medications or interventions intended to treat infantile colic. Caregivers were instructed to maintain their usual feeding practices throughout the study period.

Infant feeding characteristics were assessed at baseline and during follow-up. The proportion of breast milk in each infant’s daily milk intake was distinguished from the proportion of infants classified as breastfed at each assessment. According to the study protocol, eligible infants were required to be exclusively or predominantly breastfed, with breast milk accounting for more than 50% of feeding. Feeding patterns and the reported proportion of breast milk intake were recorded throughout the study.

#### 2.2.1. Randomization

Following confirmation of eligibility after the screening period, participants were randomly assigned in a 1:1 ratio to receive either the probiotic formulation containing *Limosilactobacillus reuteri* DSM 17938 and *Limosilactobacillus reuteri* DSM 32846 (*L. reuteri* BG-R46™) or an identical placebo for 21 days. Randomisation codes were generated before study initiation, and each participant was assigned a unique randomisation number corresponding to the allocated study product. Randomisation was completed before initiation of study treatment and served as the basis for treatment assignment throughout the trial.

#### 2.2.2. Allocation Concealment

Allocation concealment was ensured through the use of sequentially assigned study products labelled only with the study code and participant randomization number. Active treatment and placebo were identical in appearance, packaging, labelling and administration schedule. Study product labelling was performed preventing investigators and study personnel from identifying treatment allocation before database lock.

#### 2.2.3. Blinding

The study was conducted using a double-blind design. Participants’ parents or legal guardians, investigators, study personnel and the statistician remained blinded to treatment allocation throughout the study.

#### 2.2.4. Sample Size

The sample size was determined using estimates of the expected treatment effect and variability derived from previous randomized placebo-controlled trials investigating *Limosilactobacillus reuteri* DSM 17938 for infantile colic. 

Calculations were performed according to the approach described by Lachin for parallel-group clinical trials, assuming a two-sided α level of 0.05, a statistical power of 90%, and a projected dropout rate of 10%.

Under these assumptions, a total sample size of 50 randomized infants (25 per treatment group) was considered sufficient to detect a clinically meaningful difference in the primary endpoint at Day 7.

#### 2.2.5. Intervention

Participants received five drops once daily for 21 consecutive days. The active oil-based formulation contained *Limosilactobacillus reuteri* DSM 17938 and *L. reuteri* BG-R46™ at a total daily dose of 2 × 10^8^ colony-forming units (CFU), comprising 1 × 10^8^ CFU of DSM 17938 and 1 × 10^8^ CFU of BG-R46™, corresponding to a strain ratio of 1:1. The placebo consisted of the identical oil matrix without viable probiotic bacteria. Caregivers were instructed to shake the bottle, dispense five drops onto a spoon, and administer the product in association with the morning feeding at approximately the same time each day. Both products were manufactured by BioGaia Production AB (Eslöv, Sweden), supplied in identical containers, and stored at 2–8 °C.

#### 2.2.6. Statistical Analysis

Statistical analyses were performed using Microsoft Excel Office 365 and StatXact version 12.0. Continuous variables were summarized using the number of observations, mean, standard deviation (SD), median, minimum, and maximum, while categorical variables were summarized as frequencies and percentages. Baseline characteristics were compared between treatment groups using the two-sided Wilcoxon rank-sum test for continuous variables and Fisher’s exact test for categorical variables.

The prespecified primary endpoint was the change from baseline to Day 7 in total daily crying and fussing time, defined as the sum of the time recorded as awake crying, awake fussing, and inconsolable crying in the Baby’s Day Diary^®^. The primary analysis was performed in the intention-to-treat (ITT) population using the two-sided Wilcoxon rank-sum test to compare changes from baseline between the probiotic and placebo groups.

Secondary continuous outcomes, including crying and fussing duration, crying duration, sleeping time and maternal Edinburgh Postnatal Depression Scale (EPDS) scores, were summarized by treatment group and assessment time point. Changes from baseline were compared between groups using the two-sided Wilcoxon rank-sum test. Responder proportions were summarized with exact 95% confidence intervals calculated using the Clopper–Pearson method. Safety outcomes were analyzed descriptively.

All statistical tests were two-sided, with a significance level of 0.05. No adjustment for multiple comparisons was applied because secondary outcome analyses were considered exploratory. Accordingly, *p*-values for secondary outcomes were interpreted descriptively and were not intended to establish confirmatory evidence of efficacy.

#### 2.2.7. Outcome Measures

To facilitate the clinical interpretation of treatment effects, study outcomes were organized into three complementary clinical domains reflecting different dimensions of infantile colic.

### 2.3. Infant Distress

Infant crying and fussing were assessed using the Baby’s Day Diary^®^, a caregiver-completed diary used to record the duration of infant behavioral states over a 24-h period. Diary-based assessments of infant crying and fussing have been evaluated in previous methodological studies [24]. In the present trial, caregivers completed the diary at baseline, Day 7, and Day 21. The prespecified primary endpoint was the change from baseline to Day 7 in total daily infant distress time, calculated as the sum of awake fussing, crying, and inconsolable crying, expressed in minutes per day.

### 2.4. Infant Regulation

The second clinical domain evaluated infant behavioural regulation, reflecting the infant’s ability to maintain calm behavioural states throughout the day. This domain included daily sleeping time, awake and content behaviour, awake fussing and inconsolable crying, all quantified for the baseline, day 7, and day 21 using the Baby’s Day Diary^®^ These variables were considered exploratory outcomes describing behavioural regulation beyond crying duration alone.

### 2.5. Maternal Outcomes

The third clinical domain evaluated the impact of treatment on the infant–caregiver dyad. Maternal depressive symptoms were assessed using the Italian-language version of the Edinburgh Postnatal Depression Scale (EPDS), a 10-item self-report questionnaire designed to assess depressive symptoms during the postnatal period. Each item is scored from 0 to 3, yielding a total score ranging from 0 to 30, with higher scores indicating greater depressive symptom severity. The questionnaire was completed by the infants’ mothers at baseline and at the follow-up visits corresponding to Days 7 and 21 of treatment. The prespecified outcome was the change in the EPDS total score from baseline. No diagnostic cut-off was applied, as the scale was analyzed as a continuous outcome rather than used to classify mothers according to depression risk. A total score was calculated only when all 10 items were completed [25,26].

### 2.6. Safety Assessment

Safety was assessed throughout the study by recording adverse events (AEs) and serious adverse events (SAEs), as defined in the study protocol. Adverse events were evaluated for severity and relationship to the investigational product by the investigator and were summarized descriptively for all participants who received at least one dose of the study product.

## 3. Results

### 3.1. Study Population

**Figure 1:**
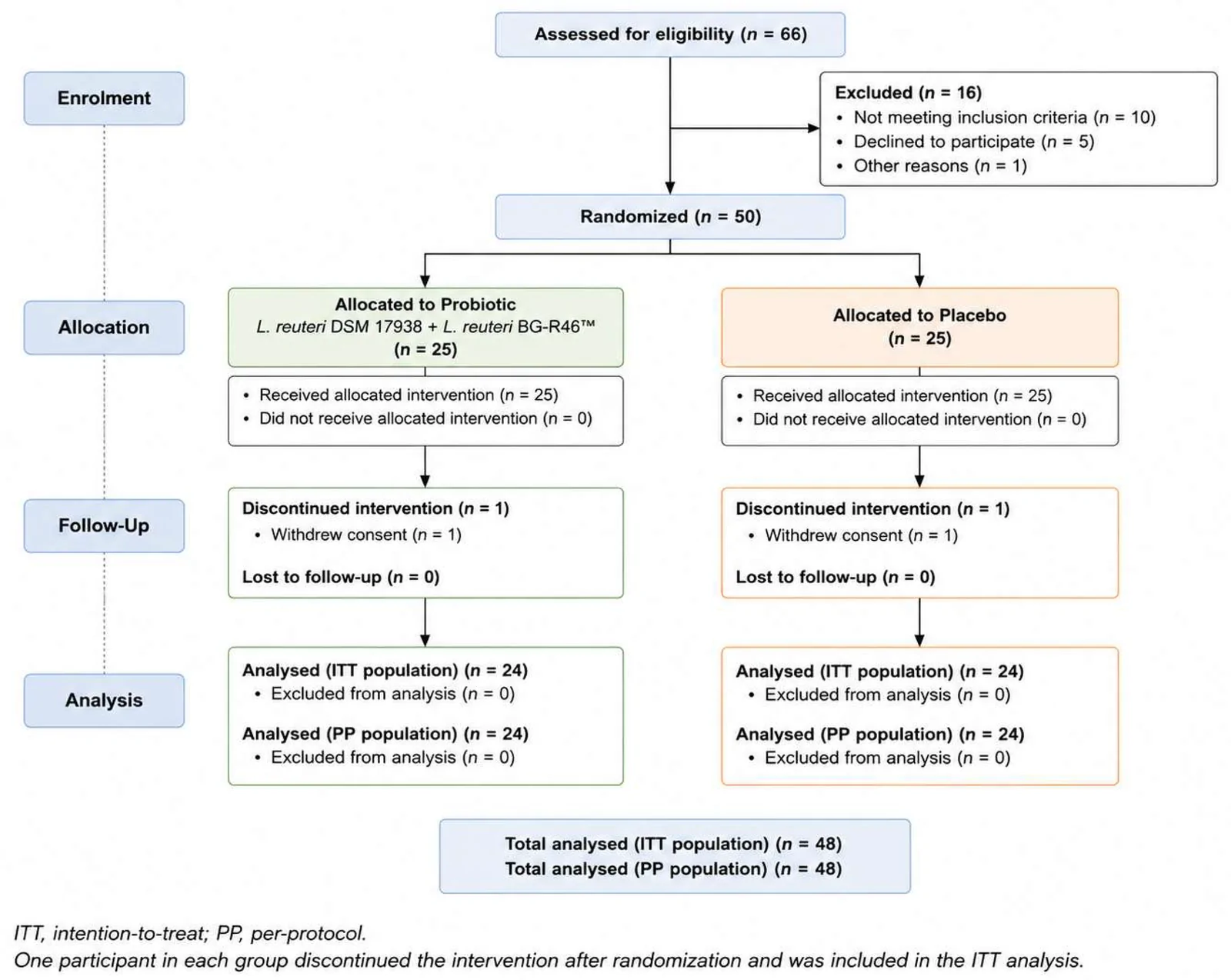
CONSORT flow diagram showing participant screening, randomization, allocation, follow-up, and inclusion in the intention-to-treat (ITT) and per-protocol (PP) analysis populations.

Between June 2025 and February 2026, 50 infants were enrolled, randomized, and received at least one dose of study product. Following verification of eligibility criteria, 48 infants were included in both the intention-to-treat (ITT) and per-protocol (PP) populations, with 24 participants allocated to each treatment group. No participant included in the ITT population was excluded from the PP analysis, reflecting excellent treatment compliance and protocol adherence throughout the intervention. A CONSORT flow diagram illustrating participant enrolment, allocation, follow-up, and analysis is presented in Figure 1 [27].

#### 3.1.1. Baseline Characteristics

The two treatment groups were comparable at baseline (Table 1). Mean age at enrolment was 41.8 ± 13.8 days in the probiotic group and 34.7 ± 6.8 days in the placebo group, with no statistically significant difference (*p* = 0.073). Sex distribution was balanced, with males accounting for 46% of participants in the probiotic group and 54% in the placebo group (*p* = 0.773). These findings indicate successful randomization without clinically relevant baseline imbalances.

#### 3.1.2. Infant Distress Time

The primary endpoint was the change from baseline to Day 7 in total infant distress time, calculated as the sum of awake fussing, crying, and inconsolable crying recorded in the Baby’s Day Diary^®^.

Baseline total infant distress time was comparable between treatment groups, averaging 545.4 ± 150.1 min/day in the probiotic group and 527.5 ± 84.3 min/day in the placebo group (*p* = 0.763).

After 7 days of supplementation, mean infant distress time decreased to 309.4 ± 146.8 min/day in infants receiving the probiotic formulation, compared with 460.8 ± 119.3 min/day in the placebo group (*p* < 0.001).

This corresponded to a mean reduction from baseline of 236.0 ± 111.5 min/day in the probiotic group versus 66.7 ± 100.1 min/day in the placebo group, with the between-group difference reaching statistical significance (*p* < 0.001).

**Figure 2:**
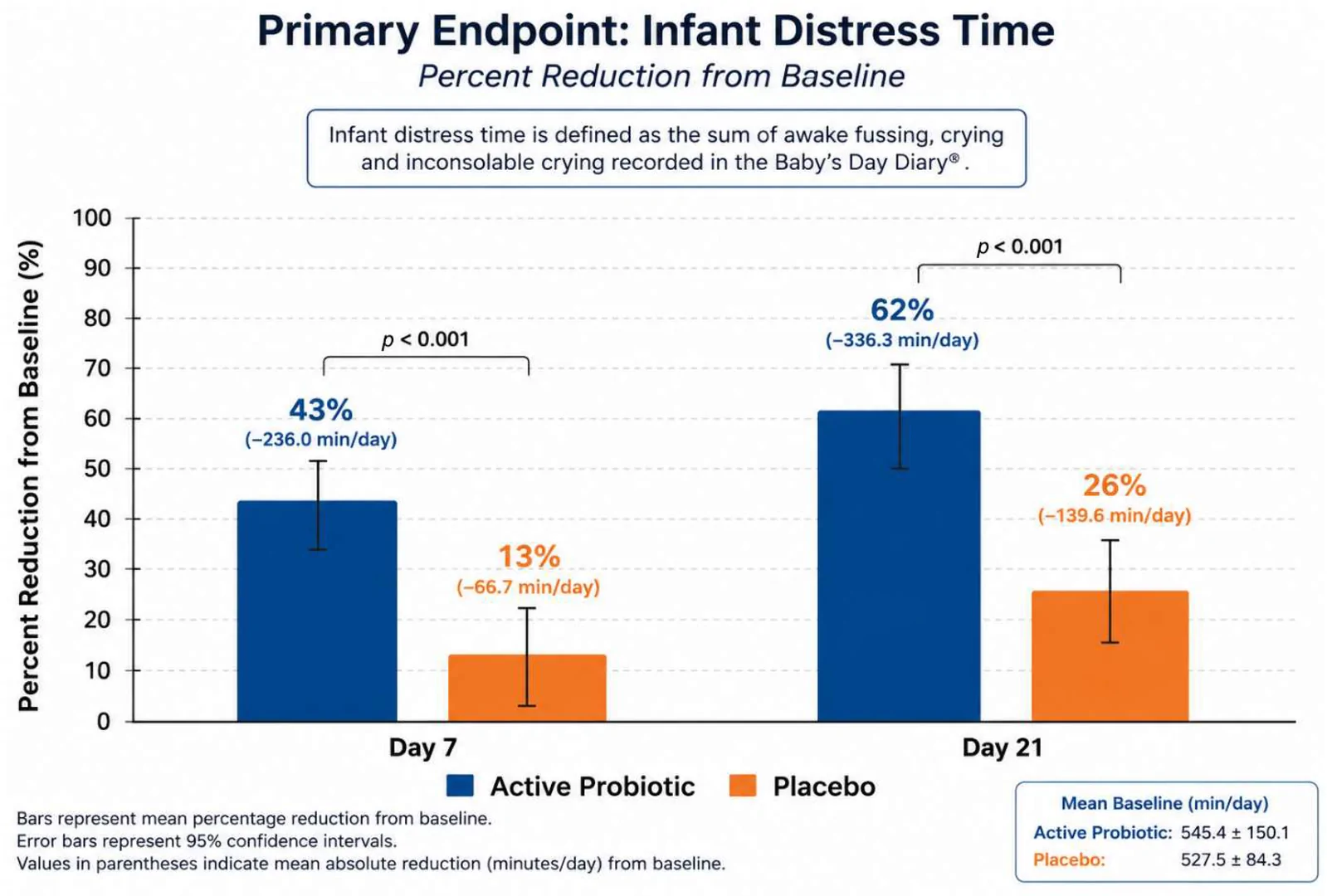
Percentage reduction from baseline in total daily infant distress time at Days 7 and 21 in the probiotic and placebo groups. Total infant distress time was defined as the sum of awake fussing, crying, and inconsolable crying recorded using the Baby’s Day Diary^®^.

Total infant distress time decreased by 43% after only 7 days in the probiotic group, compared with 13% in the placebo group, and by 62% versus 26% at Day 21 (Figure 2).

#### 3.1.3. Infant Regulation

##### Sleeping Behaviour

Daily sleep duration increased progressively during follow-up in both treatment groups. In infants receiving the probiotic formulation, mean sleeping time increased from 696.7 ± 244.3 min/day at baseline to 771.3 ± 142.1 min/day after 7 days and 793.8 ± 156.0 min/day after 21 days. In contrast, the placebo group showed a smaller improvement, with sleeping time changing from 732.5 ± 171.0 min/day at baseline to 780.8 ± 83.2 min/day at Day 7 and 737.5 ± 173.3 min/day at Day 21. Although changes from baseline were comparable between groups, infants receiving probiotics slept significantly longer than placebo-treated infants at Day 21 (*p* = 0.024).

##### Behavioural Regulation

Probiotic supplementation was associated with a progressive improvement in behavioural regulation. Time spent awake and content increased from 103.8 ± 58.4 min/day at baseline to 209.2 ± 61.8 min/day at Day 21, compared with an increase from 108.8 ± 62.1 to 163.6 ± 130.4 min/day in the placebo group. The difference between groups reached statistical significance at Day 21 (*p* = 0.001), and the improvement from baseline was significantly greater in the probiotic group (*p* = 0.021).

Conversely, awake fussing progressively declined following probiotic supplementation, decreasing from 172.5 ± 90.5 to 125.0 ± 101.4 min/day, whereas placebo-treated infants showed only a modest reduction (143.8 ± 46.0 to 123.8 ± 46.3 min/day). Absolute values differed significantly between treatment groups at Day 21 (*p* = 0.008), although changes from baseline were not significantly different.

**Figure 3:**
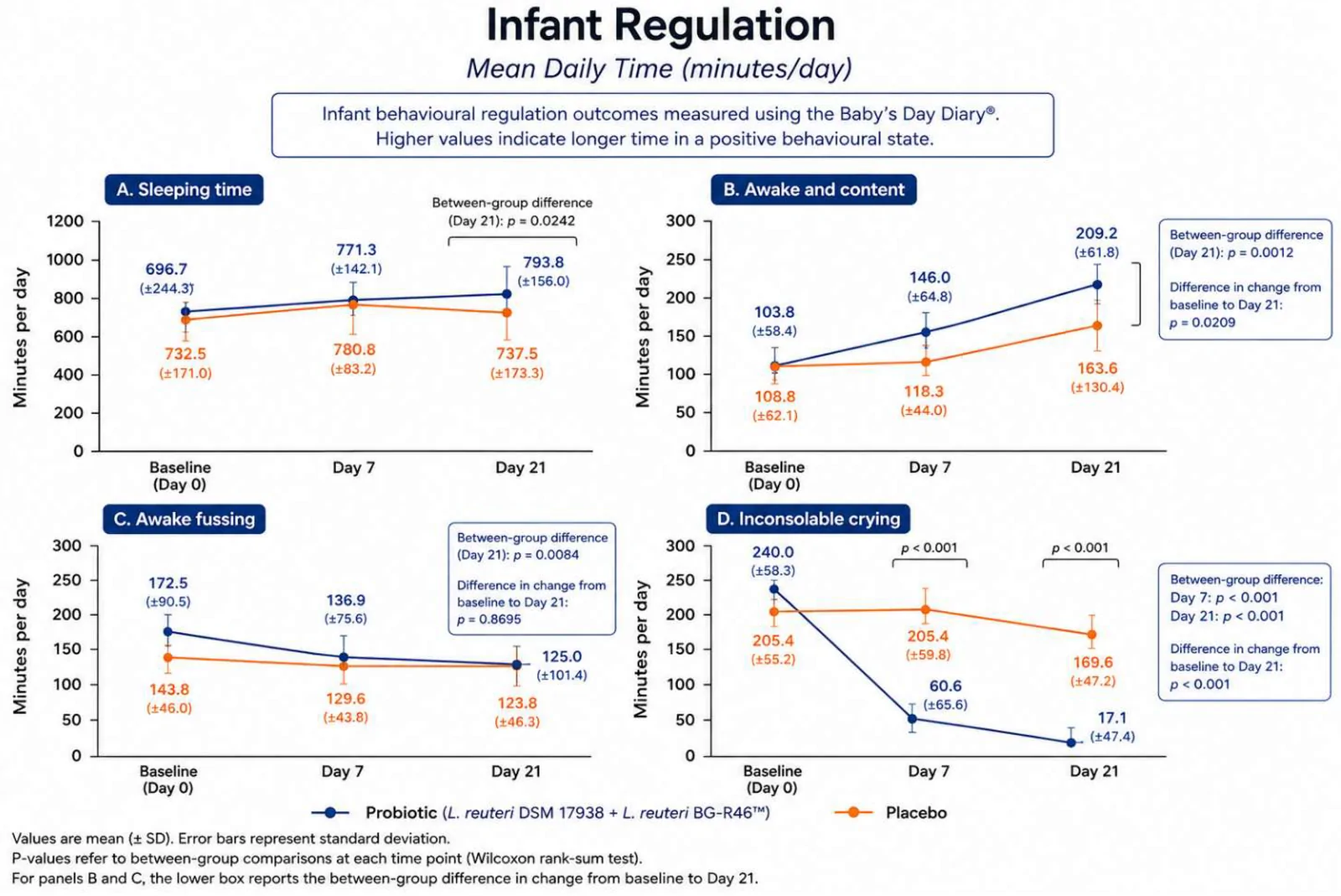
Changes in infant behavioral outcomes from baseline to Days 7 and 21 in the probiotic and placebo groups: (**A**) daily sleep duration; (**B**) awake and content time; (**C**) awake fussing time; (**D**) inconsolable crying time. Data are expressed as mean ± standard deviation (SD).

The most pronounced behavioural effect concerned inconsolable crying. Mean duration decreased dramatically from 240.0 ± 58.3 min/day at baseline to 60.6 ± 65.6 min/day after one week and to only 17.1 ± 47.4 min/day at Day 21 in the probiotic group. In comparison, placebo-treated infants still experienced 205.4 ± 55.2 min/day at Day 7 and 169.6 ± 47.2 min/day at Day 21. Between-group differences were highly significant at both time points (both *p* < 0.001), indicating a marked reduction in episodes of difficult-to-soothe crying following probiotic supplementation (Figure 3).

Responder status was defined as a reduction of at least 50% from baseline in the combined duration of awake fussing and crying, excluding the separately recorded category of inconsolable crying. This responder definition therefore differed from the composite primary endpoint, which included inconsolable crying.

At Day 7, 2/24 infants (8.3%) in the probiotic group and 0/24 (0%) in the placebo group met the responder criterion (Fisher’s exact test, *p* = 0.4894). At Day 21, the corresponding proportions were 6/24 (25.0%) and 3/24 (12.5%), respectively (*p* = 0.4614). Neither between-group difference was statistically significant.

#### 3.1.4. Maternal Outcomes

Maternal emotional well-being was assessed using the Edinburgh Postnatal Depression Scale. At baseline, mean EPDS scores were 14.41 ± 3.20 in the probiotic group and 13.20 ± 4.73 in the placebo group, with no significant between-group difference (*p* = 0.8657).

At Day 7, mean scores were 15.00 ± 5.12 in the probiotic group and 12.76 ± 5.12 in the placebo group (*p* = 0.1354). The mean change from baseline was +0.75 ± 3.61 in the probiotic group and −0.47 ± 3.20 in the placebo group, with no significant between-group difference (*p* = 0.1619). At Day 21, mean EPDS scores were 15.29 ± 3.16 in the probiotic group and 11.00 ± 6.25 in the placebo group. The between-group comparison approached, but did not reach, statistical significance (*p* = 0.0518). Changes from baseline were +0.86 ± 3.93 in the probiotic group and −1.94 ± 4.64 in the placebo group; this difference was not statistically significant (*p* = 0.0706).

#### 3.1.5. Safety

The investigational product was well tolerated throughout the study. No serious adverse events related to the study product were reported. No participant discontinued treatment because of treatment-related adverse events. Overall, the safety profile of the probiotic formulation was comparable to that of placebo.

## 4. Discussion

The present randomized, double-blind, placebo-controlled study demonstrated that supplementation with a probiotic formulation containing *Limosilactobacillus reuteri* DSM 17938 and the novel *L. reuteri* BG-R46™ strain significantly improved symptoms of infant distress in predominantly breastfed infants. Compared with placebo, probiotic supplementation resulted in an earlier and greater reduction in crying and fussing behaviour, accompanied by improvements in sleep duration. The formulation was also well tolerated, with no clinically relevant safety concerns observed during the intervention. These findings support the potential of probiotic modulation in infants with functional crying disorders and extend the current evidence available for *L. reuteri*-based interventions.

Evidence for DSM 17938 is generally favourable in breastfed infants, although findings have varied according to feeding pattern, study population, and trial methodology. The pivotal randomized trial by Savino and colleagues first reported a significant reduction in crying time after three weeks of supplementation in exclusively breastfed infants, while also suggesting that clinical improvement was associated with modifications of the intestinal microbiota. Subsequently, additional randomized controlled trials confirmed these observations, leading to meta-analyses demonstrating that DSM 17938 approximately doubles the probability of treatment success compared with placebo, particularly among breastfed infants [17,28,29,30,31,32].

Our findings are remarkably consistent with this body of evidence. A significant reduction in the primary endpoint was observed as early as Day 7, with further improvement at Day 21. Although this early response is clinically relevant, the absence of a DSM 17938 monotherapy arm prevents any conclusion regarding whether the combined formulation produces a faster or greater effect than DSM 17938 alone.

Furthermore, although reductions in crying duration represent the classical outcome used in infantile colic trials, our results indicate that the benefits of probiotic supplementation extend beyond crying alone. Improvements in sleep duration, time spent awake and content, and the marked reduction in inconsolable crying collectively suggest a broader effect on behavioural regulation rather than merely shortening episodes of crying.

This broader behavioural improvement deserves particular attention because it reflects the evolving understanding of infantile colic. In fact, the Rome V framework has shifted emphasis away from rigid temporal thresholds toward a multidimensional concept of persistent infant distress, characterized by difficult-to-soothe crying, impaired behavioural regulation and substantial caregiver concern.

Within this perspective, excessive crying is no longer viewed as the disease itself but as the most visible manifestation of a broader syndrome involving the infant–caregiver dyad.

Our findings are consistent with this contemporary conceptual model. In addition to reducing crying, probiotic supplementation improved behavioural regulation, as reflected by less inconsolable crying and longer periods during which infants remained awake and content. These outcomes may better capture clinically meaningful improvement than crying duration alone.

The relatively low responder proportions, despite the substantial reduction in the composite primary endpoint, should be interpreted in light of the different outcome definitions. The primary endpoint included awake fussing, crying, and inconsolable crying, whereas the prespecified responder analysis was based on a reduction of at least 50% in awake fussing and crying without the separately recorded inconsolable crying component. Consequently, the two analyses capture different aspects of infant distress and are not directly interchangeable.

Furthermore, dichotomizing a continuous outcome at a 50% improvement threshold may obscure clinically relevant reductions that do not meet the responder criterion. The small sample size also limited the statistical precision of comparisons between responder proportions. The responder findings should therefore be considered exploratory and should not be interpreted as confirming a treatment effect on the probability of achieving the prespecified response threshold.

No statistically significant between-group difference was observed in the change in maternal EPDS scores. Therefore, the present findings do not support a beneficial effect of the probiotic formulation on maternal emotional well-being, and this exploratory outcome should be interpreted cautiously. Furthermore, no dedicated instrument for maternal anxiety or parenting-specific stress, such as the State–Trait Anxiety Inventory (STAI) or Parenting Stress Index (PSI), was administered. Consequently, the present study cannot determine whether the intervention influenced these important dimensions of caregiver distress. Future trials should incorporate validated multidimensional measures of maternal anxiety, depressive symptoms, parenting stress, and family functioning to provide a more comprehensive assessment of the infant–caregiver dyad.

The present study has several strengths. Its randomized, double-blind, placebo-controlled design minimizes selection and observation bias, while predefined statistical analyses and evaluation in both intention-to-treat and per-protocol populations enhance the robustness of the findings.

Some limitations should also be acknowledged. An important structural limitation of the present study is the absence of a comparator arm receiving *L. reuteri* DSM 17938 alone. Consequently, although the combined formulation demonstrated efficacy compared with placebo, the study cannot establish whether BG-R46™ provides additional, additive, or synergistic clinical benefits, nor whether the combination accelerates symptom improvement relative to DSM 17938 monotherapy. Furthermore, the relatively small sample size may limit the generalizability of the findings. The study population consisted predominantly of breastfed infants, consistent with the population in which DSM 17938 has previously shown the greatest efficacy but limiting extrapolation to formula-fed infants. No microbiome analyses or inflammatory biomarkers were performed; therefore, the biological mechanisms underlying the observed clinical improvements remain speculative. The proposed complementary immunomodulatory mechanisms are derived from preclinical evidence and were not directly investigated in this trial. Finally, because the intervention combined DSM 17938 with BG-R46™, the individual contribution of the novel strain cannot be determined from the present study. Future randomized studies including both single-strain and combination treatment arms, together with mechanistic assessments, are required to clarify the specific contribution of BG-R46™. In particular, studies including separate DSM 17938, BG-R46™, combination, and placebo arms would help determine whether the combined formulation provides additional benefits beyond those of the individual strains. The integration of microbiome profiling, inflammatory biomarkers, and metabolomic analyses could also help elucidate the biological mechanisms underlying the observed clinical effects. Although previous research has suggested a potential preventive role for early-life probiotic supplementation, the present study was not designed to investigate prevention, and any prophylactic application of this formulation remains speculative and requires dedicated randomized trials.

Although the present trial was designed to evaluate treatment rather than prevention, the observed efficacy of the combination of *L. reuteri* DSM 17938 and BG-R46™ raises the hypothesis that modulation of the infant gut microbiota at an early stage could also influence the natural history of functional gastrointestinal disorders. A previous preventive study performed with *L. reuteri* DSM 17938 has suggested that early probiotic supplementation may reduce the incidence of functional gastrointestinal symptoms during infancy [33], while the long-term follow-up has explored whether modulation of the neonatal microbiota may influence the later development of disorders of gut–brain interaction [34]. Taken together, these observations support the rationale for future prospective studies evaluating whether multi-strain *L. reuteri* formulations could be used not only therapeutically but also prophylactically in infants identified as being at increased risk of functional gastrointestinal disorders [35].

## 5. Conclusions

In this randomized, double-blind, placebo-controlled trial, supplementation with a formulation containing *Limosilactobacillus reuteri* DSM 17938 and BG-R46™ significantly reduced total daily crying and fussing time at Day 7 compared with placebo in infants with infantile colic. Further reductions were observed at Day 21, together with improvements in selected exploratory measures of infant behavioral regulation. The formulation was well tolerated during the study period. These findings provide preliminary clinical evidence supporting the efficacy of the combined formulation, although larger randomized trials are needed to confirm its effects and establish the specific contribution of BG-R46™.

## Figures and Tables

**Table 1:** Baseline demographic and clinical characteristics of infants randomized to the probiotic and placebo groups.

Characteristic	Probiotic (*n* = 24)	Placebo (*n* = 24)	*p*
Age, days	41.8 ± 13.8	34.7 ± 6.8	0.0726
Male sex, *n* (%)	11 (45.8)	13 (54.2)	0.7732
Breastfed at Day 7, *n* (%)	14 (58.3)	14 (58.3)	1.0000
Baseline infant distress time (min/day)	545.4 ± 150.1	527.5 ± 84.3	0.7626

## Data Availability

The datasets generated and analysed during the current study are available from the corresponding author upon reasonable request.

## Abbreviations

AE, adverse event; EPDS, Edinburgh Postnatal Depression Scale; FGID, functional gastrointestinal disorder; ITT, intention to treat; PP, per protocol; SAE, serious adverse event.
